# Crystallographic characterization of a tri-Asp metal-binding site at the three-fold symmetry axis of LarE

**DOI:** 10.1038/s41598-020-62847-6

**Published:** 2020-04-02

**Authors:** Matthias Fellner, Kamren G. Huizenga, Robert P. Hausinger, Jian Hu

**Affiliations:** 1 Biochemistry, University of Otago, Dunedin, Otago 9054 New Zealand; 2 Biochemistry and Molecular Biology, Michigan State University, 603 Wilson Road, East Lansing, Michigan 48824 USA; 3 Microbiology and Molecular Genetics, Michigan State University, 567 Wilson Rd, East Lansing, Michigan 48824 USA; 4 Chemistry, Michigan State University, 578S Shaw Ln, East Lansing, Michigan 48824 USA

**Keywords:** Biochemistry, Structural biology

## Abstract

Detailed crystallographic characterization of a tri-aspartate metal-binding site previously identified on the three-fold symmetry axis of a hexameric enzyme, LarE from *Lactobacillus plantarum*, was conducted. By screening an array of monovalent, divalent, and trivalent metal ions, we demonstrated that this metal binding site stoichiometrically binds Ca^2+^, Mn^2+^, Fe^2+^/Fe^3+^, Co^2+^, Ni^2+^, Cu^2+^, Zn^2+^, and Cd^2+^, but not monovalent metal ions, Cr^3+^, Mg^2+^, Y^3+^, Sr^2+^ or Ba^2+^. Extensive database searches resulted in only 13 similar metal binding sites in other proteins, indicative of the rareness of tri-aspartate architectures, which allows for engineering such a selective multivalent metal ion binding site into target macromolecules for structural and biophysical characterization.

## Introduction

Metals are essential components of many biological macromolecules, especially proteins. About a third^1,2^ of the protein structures in the PDB^3^ contain one or more metal atoms. The atomic structures of metal-containing cofactors and other metal-binding sites often provide insights into molecular mechanisms and/or reveal critical structural roles. Ten metal cations Mg^2+^, K^+^, Ca^2+^, Mn^2+^, Fe^2+^, Co^2+^, Ni^2+^, Cu^2+/+^, and Zn^2+^ are commonly associated with proteins^4^, but other elements also may play important roles in catalysis or are associated with toxic effects^1^. Because of their unique spectroscopic characteristics, some transition metals have been used to study structural and dynamics properties of macromolecules^5–7^.

In this study, we present a crystallographic analysis of a recently discovered^8^ tri-Asp metal-binding site on the three-fold symmetry axis of the hexameric LarE protein from *Lactobacillus plantarum*. LarE^8,9^, a member of the PP-loop ATP pyrophosphatase family^10^, in conjunction with LarB^11,12^ and LarC^13^, participates in the synthesis of the nickel-pincer nucleotide cofactor of lactate racemase^14–17^. Our crystallographic studies indicated binding specificity towards certain divalent or trivalent metal ions. An extensive database search for similar metal binding sites in other proteins resulted in only 13 hits and structural comparison among these sites revealed the uniqueness of the tri-Asp site in LarE. Given its rarity in protein structures, we propose that such a metal binding site can be engineered into macromolecules^18,19^ of interest to facilitate structural and biophysical characterization.

## Materials and methods

### Crystallization optimization of LarE

LarE from *L. plantarum* (Table S1) initially was overexpressed and purified from *Lactococcus lactis* cells containing pGIR072^8,11^. Broad screening for crystallization at 21 °C resulted in small diamond-shaped crystals (Table S2) in space groups R3 or C212121, both from the same crystallization conditions of 0.1 M imidazole, pH 7.0, 0.15 M malic acid, pH 7.0, and 22% poly(ethylene glycol) monomethyl ether 550. Both crystal forms revealed an overall hexamer and both contained a metal bound at low occupancy, but with a strong anomalous signal at a wavelength of 0.979 Å (R3) and 0.999 Å (C212121), to residue Asp231 of each of three LarE chains; we termed this site the tri-Asp metal-binding site of LarE. We added no metal during enzyme purification or crystallization, so the metal identity was unclear.

To enhance protein yield for optimizing crystallization, we switched to an *Escherichia coli* expression system (Table S1) using construct pGIR076^8^. Broad screening at 21 °C revealed several crystallization hits that were further optimized (Table S2). We previously reported two different apoprotein species (5UDQ, 5UNM), three substrate-bound forms (5UDR, 5UDS, 5UDT), and four different tri-Asp metal-bound (Mn, Fe, Ni, and Zn) structures (5UDU, 5UDV, 5UDW, and 5UDX)^8^. We present here four additional tri-Asp metal-bound (Ca, Co, Cu, and Cd) structures (6UTP, 6UTQ, 6UTR, and 6UTT). Table S3 summarizes all LarE structures.

### Analysis of LarE metal binding by crystallographic methods

#### Soaking experiments

If not stated otherwise all soaking solutions contained a metal dissolved in 50 mM ammonium sulfate, 50 mM Bis-Tris, pH 6.5, and 30.0% v/v pentaerythritol ethoxylate (15/4 EO/OH). The analyzed datasets ranged between 2.3 and 3.6 Å resolution, with the majority around 2.6 Å. After molecular replacement and one round of refinement using default parameters in Phenix^20^, the presence of a bound metal was assessed. In all cases, datasets of crystals that were not soaked also were determined in parallel to confirm the absence of a metal. Characterization of the metal-binding site by crystallography included several variations of conditions. We altered the timespans of crystal soaking with a metal solution (0.5, 5, 30, 60 min, and 22 h) using 3.8 mM FeSO_4_. As datasets from all time points showed similar metal electron density, all reported soaking experiments were performed for one hour, if not stated otherwise. We examined a wide range of metal ions to investigate the metal specificity of the tri-Asp binding site at metal concentrations of 3.8 mM, unless stated otherwise.

#### Crystallization, data collection and structure determination of Ca, Co, Cu, and Cd bound structures

For crystallization, 5 μL (0.6 μL for Co) of ~25 mg/ml LarE (100 mM Tris-HCl, pH 7.5, 300 mM NaCl) were mixed with 5 μL (1.2 μL for Co) of reservoir solution. The hanging drop reservoir contained 100 μL of 30% (25% for Co) v/v pentaerythritol ethoxylate (15/4 EO/OH), 50 mM Bis-Tris pH 6.5 (50 mM MOPS pH 7.0 for Co), and 100 mM ammonium sulfate. The formed crystals were soaked 60 min in 3.8 mM calcium chloride or copper(II) sulfate or cadmium chloride dissolved in 30% v/v pentaerythritol ethoxylate (15/4 EO/OH), 50 mM Bis-Tris, pH 6.5, and 50 mM ammonium sulfate. The cobalt sulfate soaking time was reduced to 5 min as it appeared to damage the crystals rapidly; its final concentration may differ from 3.8 mM due to the low solubility of the metal salt.

Data sets were collected at the Advanced Photon Source LS-CAT beamlines (21-ID-D). As the Co soaked crystal diffracted to lower resolution than the other crystals, data collection was done at the Co K-edge at 1.600 Å to optimize the anomalous signal. The other datasets were collected at the default wavelengths at the respective beamlines (0.979 Å and 1.127 Å), which still give reasonable anomalous signal for these elements. Datasets were processed with xds^21^, with merging and scaling done using aimless^22^. Phenix Phaser molecular replacement^20^ used the wild-type apoprotein model 5UDQ. Model building and refinement were conducted in Coot^23^ and Phenix^20^. Simulated annealing composite-omit maps for the entire molecule were created in Phenix. The original experimental data created by aimless and the final pdb file without further refinement were used as input files. Crystallographic statistics are listed in Tables 1 and 2. UCSF Chimera^24^ was used to create structure figures.

**Table 1:** Data collection and processing.

	Calcium bound	Cobalt bound	Copper bound	Cadmium bound
Diffraction source	APS 21-ID-D	APS 21-ID-D	APS 21-ID-D	APS 21-ID-D
Wavelength (Å)	0.979	1.600	1.127	1.127
Detector	DECTRIS EIGER X 9 M	DECTRIS EIGER X 9 M	DECTRIS EIGER X 9 M	DECTRIS EIGER X 9 M
Space group	P 4_1_ 2 2	P 4_1_ 2 2	P 4_1_ 2 2	P 4_1_ 2 2
a, b, c (Å)	107.7, 107.7, 320.4	106.2, 106.2, 313.6	109.0, 109.0, 323.7	107.8, 107.8, 319.6
α, β, γ (°)	90, 90, 90	90, 90, 90	90, 90, 90	90, 90, 90
Resolution range (Å)	48.15–2.49 (2.55–2.49)	87.93–3.55 (3.83–3.55)	48.74–2.41 (2.46–2.41)	48.98–2.39 (2.43–2.39)
Total No. of reflections	555563 (31312)	212999 (20329)	1163885 (75724)	742009 (75461)
No. of unique reflections	66185 (3834)	43827 (3838)	60947 (4095)	41119 (4302)
Completeness (%)	98.5 (86.6)	89.6 (84.4)	99.5 (93.1)	99.3 (97.5)
Redundancy	8.4 (8.2)	10.5 (11.4)	15.4 (14.9)	9.8 (9.6)
〈 *I*/σ(*I*)〉	18.2 (2.0)	9.0 (3.2)	15.6 (1.9)	17.8 (2.1)
CC_1/2_	0.999 (0.758)	0.994 (0.914)	0.999 (0.705)	0.999 (0.682)
*R*_merge._	0.069 (0.866)	0.218 (0.818)	0.102 (1.245)	0.069 (1.188)
*R*_p.i.m._	0.036 (0.452)	0.091 (0.322)	0.038 (0.467)	0.033 (0.573)

Values for the outer shell are given in parentheses.

**Table 2:** Structure solution and refinement.

	Calcium bound	Cobalt bound	Copper bound	Cadmium bound
Resolution range (Å)	38.49–2.49 (2.52–2.49)	87.93–3.55 (3.64–3.55)	48.35–2.41 (2.41–2.44)	37.88–2.39 (2.41–2.39)
Final *R*_cryst_	0.202 (0.317)	0.231 (0.283)	0.208 (0.337)	0.213 (0.312)
Final *R*_free_	0.256 (0.357)	0.298 (0.311)	0.253 (0.380)	0.254 (0.326)
Metal atoms	2	6	2	7
Phosphate molecules	6	6	6	6
Sulfate molecules	6	10	5	7
Water	87	13	122	175
R.m.s. deviations				
Bonds (Å)	0.008	0.010	0.009	0.008
Angles (°)	0.999	1.126	1.107	1.1050
Average *B* factors (Å^2^)	66.8	74.4	65.6	67.8
Metal	64.8	62.74	66.2	91.9
Phosphate	59.4	71.7	59.6	64.1
Sulfate	76.6	74.5	71.1	75.0
Ramachandran plot				
Most favoured (%)	97.50	95.44	96.94	98.0
Outlier (%)	0.0	0.0	0.0	0.0

Values for the outer shell are given in parentheses.

#### Co-crystallization experiments

LarE was screened for co-crystallization of components in the Hampton Additive Screen (HR2-428) using two different conditions (Table S2) at the recommended concentrations (5 μL protein plus 1 μL additive plus 4 μL reservoir).

## Results and Discussion

### LarE contains an unusual tri-Asp metal-binding site

**Figure 1:**
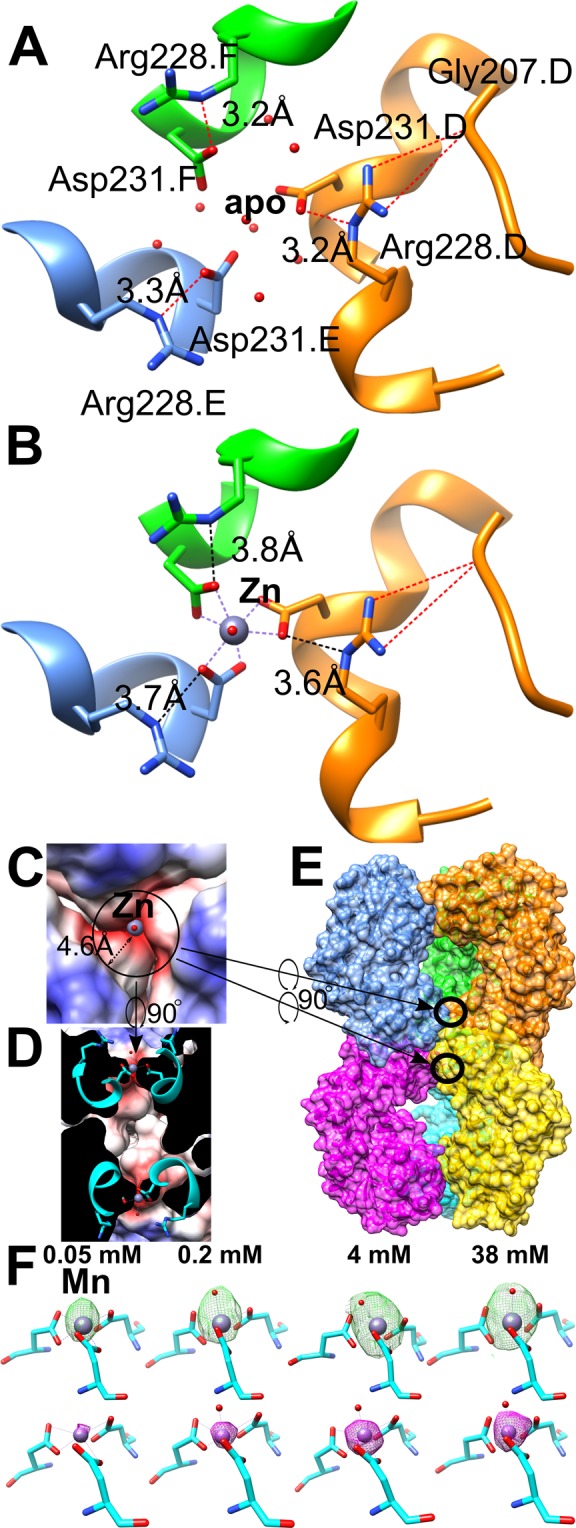
Tri-Asp metal binding site in LarE. (**A**) Close-up of the tri-Asp site in the apoprotein structure (PDB ID 5UDQ) filled with water molecules, illustrated as red spheres. Hydrogen bonds involving Arg228 are shown as red dashed lines, with the distances of the bonds indicated. The letter after the residue name and number indicates the protein chain. (**B**) Same view upon metal binding, illustrated for the Zn bound structure (PDB ID 5UDX). (**C**) Same top view and structure as B with electrostatic potential shown. (**D**) 90 degree turned cross-sectional view of the tri-Asp site within the hexamer. (**E**) Zoomed out view of D with chains surface colored individually. The two tri-Asp sites are circled. (**F**) Changes of the *mFo-DFc* electron density map at the tri-Asp site corresponding to different soaking concentrations of Mn. The maps are shown in green at 3 σ after one round of refining the molecular replacement solution. The models shown have been refined with one Mn and one water molecule; the Mn atom is shown in violet and the water oxygen atom in red. Below is shown the corresponding anomalous signal map in magenta at 6 σ.

Crystals of *E. coli*-derived LarE protein were hexameric and possessed metals bound to the tri-Asp sites when they had been subjected to size-exclusion chromatography, whereas the crystals were free of metal when the protein was not subjected to the chromatography step (Fig. 1A). We reason that the tri-Asp site captures low levels of contaminating metal ions, most likely Ni, Co, or Cd, from the Superdex 200 resin used for other protein purifications in the laboratory. This result implies that the trimer is capable of binding metals with high affinity in solution. Omission of the size-exclusion step from all further LarE purifications used for crystallization experiments had no effect on crystal growth. We also observed weak binding to the tri-Asp site by unidentified metal(s) in fully-grown crystals that had been left untouched for months. These observations indicate that the tri-Asp site is able to extract low levels of metal impurities from different environments. In the following crystal soaking experiments, we excluded the crystals older than two weeks to avoid spontaneous metal enrichment at the tri-Asp site.

In the apoprotein structure (Fig. 1A) the tri-Asp site is pre-formed to receive a metal ion by a hydrogen bond network consisting of Asp231, Arg228, Gly207, and several water molecules filling the site. Notably, the arginine residues form a positively charged ring around the tri-Asp site, limiting the metal-Asp interactions along the three-fold symmetry axis and likely responsible for only one metal ion being captured by these residues. Metal binding occurs with displacement of the water molecules (Fig. 1B). The Asp231 side chains move closer together to chelate the metal, in the process weakening the hydrogen bonds with the Arg228 side chains, which move slightly further away. Taking protonation into account, in the apoprotein the hydrated metal has to pass through a ~4.6 Å radius charged ring (Fig. 1C) introduced by the Arg residues. Arg228 itself is kept in place by hydrogen bonding to the backbone of a nearby loop containing Gly207. This interaction is nearly unchanged between the apoprotein and metal bound state (Fig. 1A,B), and overall only Asp231 and Arg228 appear to be influenced by metal binding. The charged ring may play a role in facilitating the binding of certain metals over others, as the metal has to pass by the arginine ring. This appears to be the only access towards the tri-Asp site as the head to head trimer-trimer interface blocks access from the other side in the hexamer where an inaccessible cavity is found (Fig. 1D,E). As shown in Fig. 1F, Mn^2+^ binds to this site in a concentration-dependent manner and the electron density of Mn reaches a maximum at 4 mM, and the same for the anomalous signals. Therefore, in the later experiments, the crystal soaking solutions contained approximately 4 mM metal ions of interest.

**Figure 2:**
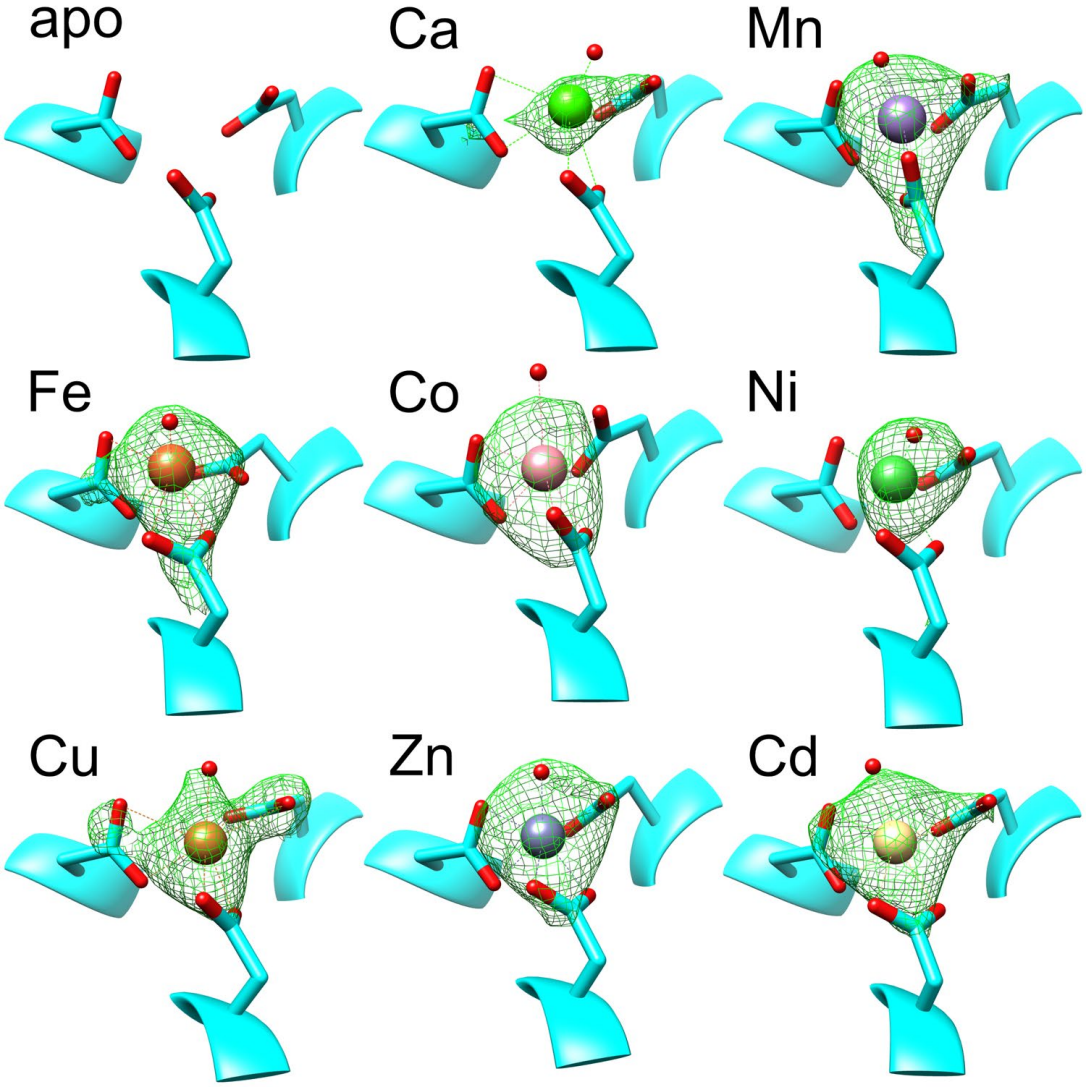
Simulated annealing composite-omit maps of tri-Asp sites in LarE structures. Shown are *2mF*_*o*_*-DF*_*c*_ maps at 3 σ within 4 Å of the metal atom (or in case of apoprotein, 2.5 Å from the Asp231 carboxyl oxygen atoms). Metals are colored using the Jmol element colors. Corresponding dataset information is listed in Table S3.

We previously reported that the tri-Asp site can bind Mn^2+^ (PDB ID code 5UDU), Fe^2+^ (5UDV), Ni^2+^ (5UDW), or Zn^2+^ (5UDX)^8^, including anomalous peak maps and element identification for Ni with datasets collected above and below the Ni K-edge. Here we examined the effect of added Ca^2+^ (6UTT), Co^2+^ (6UTP), Cu^2+^ (6UTR), and Cd^2+^ (6UTQ) (Fig. 2). In all cases, the Asp231 side chains bind the metals with bidentate coordination, with a seventh site always showing extra density that we modelled as a water molecule. Even in the case of the low resolution Co^2+^ bound dataset this extra density is observed. As 3.55 Å dataset resolution cannot justify placement of water alone we inferred the shown mode based on the observations in the other datasets. Simulated annealing composite-omit maps of all metal bound structures clearly indicate the presence of these metals when compared to the apoprotein structure (Fig. 2). The carboxyl-group of all Asp231 residues shows higher B-factors than the backbone in that region or than the metal atom itself, so we cannot rule out that our seven coordination model is an average of mixed monodentate and bidentate chelation, resulting in a lower coordination number. Nevertheless, the best interpretation of electron density is always a seven-coordinated metal atom consistent across all observed elements. Although seven-coordinated metals appear to be unusual, MetalPDB^25^ contains 1573 structures with metals having a coordination number of seven. The eight metals included in our LarE datasets account for 1239 of the seven-coordinate metal-containing protein structures.

### Crystallographic characterization of metal binding to the tri-Asp site

To probe the elemental preference of the metal-binding site, we soaked crystals with 3.8 mM of MgCl_2_, MgSO_4_, KCl, K_2_HPO_4_, Ca acetate, CaCl_2_, CrCl_3_, MnSO_4_, FeSO_4_, FeCl_3_, CoSO_4_, NiSO_4_, CuCl, CuCl_2_, ZnSO_4_, CdCl_2_, and CsCl. We observed binding to the tri-Asp site for Ca^2+^, Mn^2+^, both Fe^2+^ and Fe^3+^, Co^2+^, Ni^2+^, Cu^2+^, Zn^2+^, and Cd^2+^, whereas Mg^2+^, K^+^, Cr^3+^, Cu^+^ and Cs^+^ did not bind at that location (data not shown). The oxidation state of the metals throughout the text is inferred from the oxidation state of the reagents used. We cannot rule out changes of oxidation states after dissolving the reagents in the soaking solutions, exposure to air for an hour during soaking, or due to exposure to synchrotron radiation. For example, Fe^2+^ and Fe^3+^ cannot be differentiated based on electron density. Although Cu^+^ is readily oxidized to Cu^2+^ we saw a clear difference between Cu^+^ and Cu^2+^ soaked datasets. In general, soaking experiments reduced diffraction power and had a negative effect on merging statistics; especially the Cu and Co datasets were of worse quality than the apoprotein datasets or those for other metals like Ca. Several metal soaks (Co, Ni, Zn, and Cd) also resulted in binding of these metals to surface exposed residues. These partially disordered sites only appeared after metal soaks, showing a significant electron density peak and in some cases anomalous peaks. In most cases the surface site involved only a single histidine residue. This resulted in higher B-factors of these metals compared to the tri-Asp site. For example, the two Cd^2+^ metals (PDB ID 6UTQ) at the tri-Asp site have an average B-factor of 60.1 Å^2^, while the average of all modelled cadmium atoms in the structure is significantly higher (Table 2).

We found that the binding of most metal ions to the LarE tri-Asp site in crystal soaking experiments prevented crystal growth during co-crystallization studies. In additive screening, we were able to obtain datasets for LarE co-crystallized with 10 mM CrCl_3_, NaBr, SrCl_2_, YCl_3_, and BaCl_2_ or with 100 mM NaI and CsCl as well as with the organic compounds glutathione (1 mM), glycyl-glycyl-glycine (30 mM), glycine (100 mM), phenol (10 mM), and L-proline (10 mM). Cr^3+^ and Cs^+^ did not bind at the tri-Asp site in these datasets, confirming the soaking results. Similarly, Br^−^, Sr^2+^, Y^3+^, I^−^, and Ba^2+^, as well as the mentioned organic compounds, did not bind to LarE.

### Tri-Asp metal-binding sites at three-fold symmetry axes in other proteins

**Figure 3:**
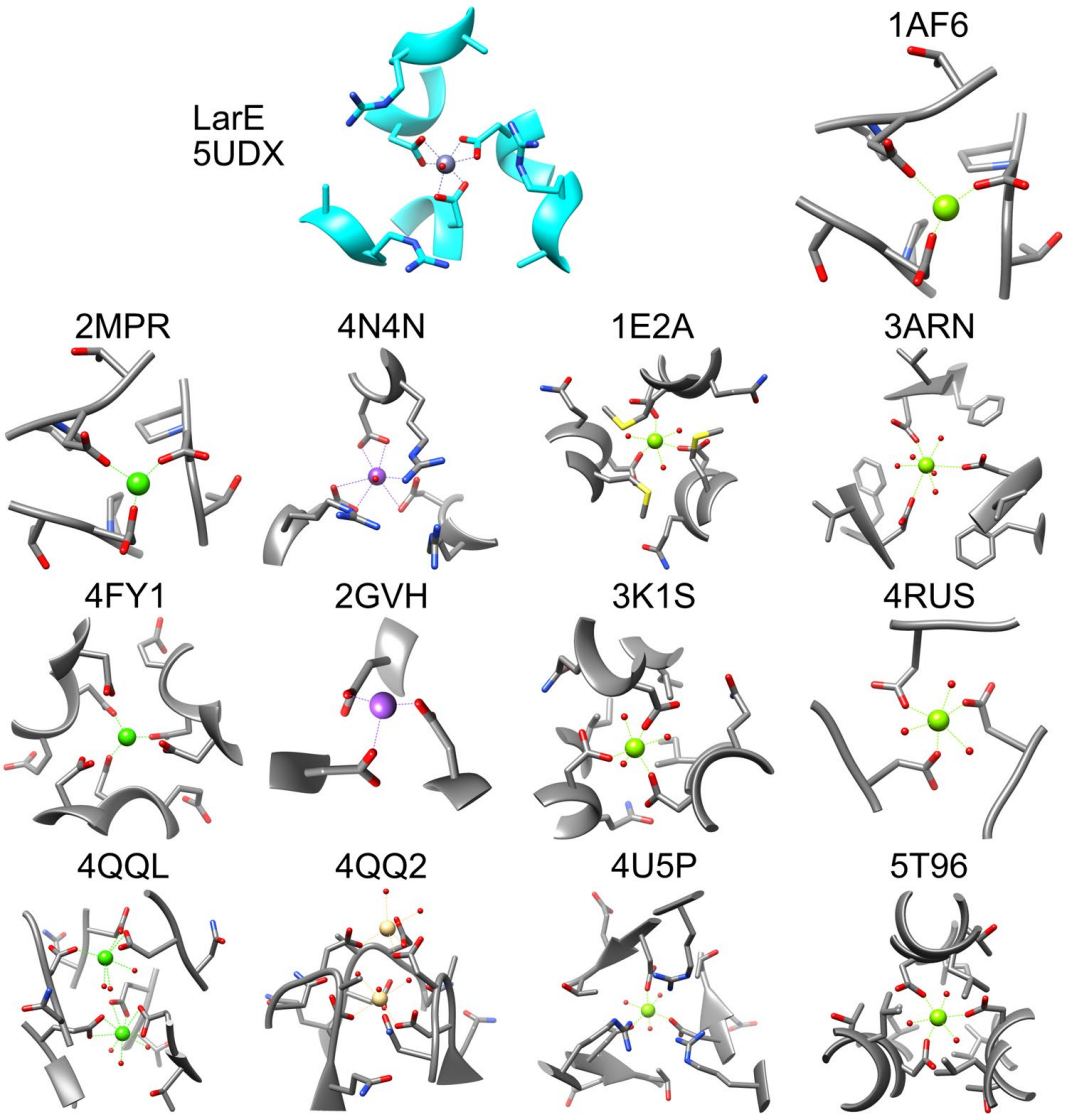
Tri-Asp metal-binding sites in proteins at three-fold symmetry axes. For LarE the Zn^2+^ bound structure 5UDX is shown. The Zn^2+^ atom is dark purple, Mg^2+^ is grass green, Ca^2+^ is green, K^+^ is purple, and Cd^2+^ is light brown. Illustrations are scaled to show all residues within 6 Å of the metal. Sites shown for *E. coli* maltoporin 1AF6^40^, *S. typhimurium* maltoporin 2MPR^41^, hydroxylamine oxidoreductase 4N4N^29^, enzyme IIA^lactose^ 1E2A^42^, deoxyuridine triphosphatase 3ARN^43^, Panicum mosaic virus 4FY1^44^, acyl-CoA hydrolase 2GVH (unpublished), PTS cellobiose specific enzyme IIA 3K1S (unpublished), carp fishelectin 4RUS^45^, C1q-like protein 4QQL and 4QQ2^46^, RhCC oxygenase 4U5P^47^, and salmon anemia virus receptor complex 5T96^48^.

We attempted to identify similar metal-binding sites in the protein databank using various webservers. MetalPDB^25^ (as of August 2019) contains 6304 protein (1736 unique sequences) structures with metal binding sites coordinated by at least three aspartate residues. 338 structures (218 unique sequences) contain sites with only three aspartate residues chelating; i.e. without any other protein residue being involved. We narrowed these hits to tri-Asp sites in proteins at three-fold symmetry axes and combined them with hits from other searches using MIPS^26^, MetalS3^27^, and Metalmine^28^ resulting in 14 sites, including LarE (Fig. 3).

Beyond the tri-Asp chelation these sites do not appear to share any nearby structure features. The sites are surrounded by either no residues (2GVH, 4RUS), hydrophobic residues (3ARN, 3K1S, 5T96), positively charged (4N4N, 4U5P) similar to LarE, polar uncharged (1AF6, 2MPR), methionines (1E2A), or negatively charged residues (4FY1, 4QQL, 4QQ2). In contrast to LarE these sites are mostly occupied by lighter elements like Na and in particular Mg. The Ca sites and the single Cd site do not share any resemblance to LarE. Lastly, nearly all structures show monodentate binding, often with three additional water molecules resulting in an octahedral coordination site.

Only one match with a similar chelation geometry to LarE was identified: *Nitrosomonas europaea* hydroxylamine oxidoreductase (Fig. 3, PDB ID 4N4N)^29^. In that case, three Asp residues bind K^+^ via bidentate coordination, with a seventh site occupied by a water molecule. Here an Arg side chain also gates one entrance side, however as the Arg residue is adjacent to the Asp residue and not one helix turn away like in LarE the gate appears to be narrower. In hydroxylamine oxidoreductase, however, the opposite side is surface exposed, while in LarE the other trimer of the hexamer blocks accessibility (Fig. 1D). The monovalent ion was not discussed in that publication and the metal speciation of that site may differ from K^+^ as the interpretation of this cation was apparently based solely on the crystallization conditions. Thus, the metal-bound tri-Asp site of LarE is rare; however, we do not know whether such sites may exist in other apoprotein structures.

Compared with the potassium-bound tri-Asp site in 4N4N (and 4N4O), the unique environment of the tri-Asp site in LarE blocks the binding of alkali cations. The back-to-back trimer arrangement allows the metal to approach only through the arginine ring, which reduces electrostatic interaction and therefore effectively prevents monovalent cation binding. The stronger coulombic force between the negatively charged sink (Fig. 1C) and the di- or tri-valent cations may overcome the repulsion imposed by the arginine ring, resulting in metal binding to the tri-Asp site strictly along the three-fold axis. However, not all of the tested multivalent cations were captured by the tri-Asp site. Multiple factors may contribute to the observed metal selectivity among the studied multivalent cations. Firstly, size matters^30^. It is shown that the metal ions with a radius greater than that of Ca^2+^ (100 pm), like Sr^2+^ or Ba^2+^ cannot bind (Table S4). Secondly, Cr^3+^ is kinetically inert in ligand exchange, and so it is not expected to bind efficiently. Thirdly, Mg^2+^ is known to exhibit a lower first stability constant for replacement of water in the aqueous ion by a ligand compared to other divalent transition metal ions in the Irving-Williams series^31,32^. A recent MD simulation study using a polarizable force field provides insights on Mg’s low affinity towards many Ca-preferred highly charged metal binding sites^33^. Crystallographically, Mg^2+^ is much lighter than the other multivalent cations studied in this work, so Mg^2+^ binding with low occupancy would not be readily detected. Although we did not see any density upon MgSO_4_ or MgCl_2_ soaking, we cannot exclude the possibility that Mg^2+^ can actually weakly bind there. One additional putative factor would be the arginine ring. Although electrically unfavorable for metal binding, arginine residues can directly coordinate transition metal ions using the nitrogen atoms in the guanidine moiety^25,34^. The hypothetical interaction between the arginine residues and “soft” metals may facilitate the latter to pass through the arginine ring, whereas the same interaction is unlikely to happen for a hard cation (Mg^2+^, Ca^2+^, Ba^2+^, Sr^2+^, and Y^3+^ studied here). Y^3+^ binding to proteins appears to be rare in general with only 41 PDB entries^3^. Also single positively-charged metals and negatively charged elements like Br^−^ and I^−^ appear to be unable to bind.

Coordination number is another factor that may play an important role in metal preference at the tri-Asp site. The metal atoms in all of our structures are hexacoordinated by carboxyl groups with a seventh coordination site always showing positive density that we interpreted as a water molecule. For small molecule complexes in the Cambridge Structural Database (CSD) the most common and second most frequent coordination numbers for Mn^2+^, Fe^2+^/^3+^, Co^2+^, Ni^2+^, Zn^2+^, and Cd^2+^ are 6 and 4; while for Ca^2+^ they are 6 and 7; and for Cu^2+^ they are 5 and 6^35^. Thus, the preference for hexacoordination generally fits with our binding at the tri-Asp site. A coordination number of 7 is only seen frequently in Ca^2+^ but does exist for the other elements in small molecule complexes within the CSD. However, the coordination number alone might not be the best predictor of binding to the LarE tri-Asp site, as the most common or second most frequent coordination number for Cr^3+^, Y^3+^ and Sr^2+^ is also 6 in the CSD; and for Ba^2+^ the most common are 7 and 8. We also investigated the coordination number of metal atoms in proteins using MetalPDB^25^ (Table S5). The results show a similar picture for these elements with the most frequent coordination number being 6 for Mn, Fe, Co, and Ni; 7 (closely followed by 6) for Ca; and 4 for Cu, Zn, and Cd (with 6 coordination being less frequently observed). Y, Sr, and Ba have a limited number of PDB entries with the majority having coordination numbers of 1–3, suggesting a lack of complete hydration, in agreement with the lack of visible water molecules in those protein structures. Cr only has 10 entries in MetalPDB, again illustrating its inert nature. Lastly Mg^2+^ in small molecules^35^ most frequently has a coordination number of 6, with 7 being rarely observed. In addition to the reasons already outlined that exclude Mg from binding, there appears to be a significant difference in the hexacoordination of Mg by carboxylate side chains compared to the other metals. In 96% of the cases for this subset of protein structures, Mg exhibits monodentate chelation by Asp/Glu residues^36^. This behavior also is illustrated for Mg atoms chelated by three Asp residues at three-fold symmetry axes (Fig. 3; PDB entries 1AF6, 1E2A, 3ARN, 3K1S, 4RUS, 4U5P, 5T96). The frequent observation of hexacoordinated Mg is achieved by the addition of three water molecules to the three amino acid side chains. In contrast to the nearly exclusive monodentate binding of Mg by carboxylates, the Ca sites in protein structures involving carboxylates are 71% monodentate and 29% bidentate^36^. This difference gives another strong argument why Mg^2+^ binding was not observed for LarE. Interestingly, when carboxyl groups are involved in chelating Zn^2+^ or Cd^2+^ in protein structures, 7 coordination (as observed in LarE) is actually the most frequently observed coordination^37^.

Additional investigation (such as metal binding to a variant with substituted Arg228) is warranted to determine the exact mechanism for metal specificity at the tri-Asp site in LarE. Nevertheless, the tri-Asp site in LarE represents an unusual metal binding site that selectively and stoichiometrically binds certain multivalent cations.

### Implications

As a D231R variant showed the same activity as wild-type protein and a similar hexameric gel filtration profile^8^, we conclude that no biological role can be established for this metal binding site based on our data. However, this rare metal binding site could be of interest to those working with small molecule mimics or for targeted protein engineering to create a multivalent metal binding site. This site, able to capture trace amounts of metal ions from solution when the protein is in its soluble or crystalline state, has been demonstrated to bind Ca^2+^, Mn^2+^, Fe^2+^/Fe^3+^, Co^2+^, Ni^2+^, Cu^2+^, Zn^2+^, and Cd^2+^, many of which have characteristic spectroscopic properties.

By engineering such a tri-Asp site into other target proteins, the binding of selected metals, such as Cu^2+^ and Ni^2+^ ^38,39^, could be used to probe protein structure and dynamics. While it is known that acidic residues bind such divalent metal cations, the distinct properties of the LarE-type metal-binding site (i.e., its ability to capture trace amounts of metal ion, its broad yet defined metal ion specificity, and its apparent uniqueness in proteins) make it an intriguing site for protein engineering, particularly at trimer interfaces. The presence of a single water molecule at the metal-binding site also raises the possibility that such engineered proteins could exhibit catalytic activities involving activation of this ligand.

## Supplementary information

Supplementary Information.

## Data Availability

The datasets generated and analysed during the current study are available in the worldwide Protein Data Bank under PDB IDs 6UTT (Ca bound), 6UTP (Co bound), 6UTR (Cu bound), and 6UTQ (Cd bound).
