# Supplementary material for: Crystallographic characterization of a tri-Asp metal-binding site at the three-fold symmetry axis of LarE
Source: Sci Rep. 2020 Apr 2;10:5830. doi: 10.1038/s41598-020-62847-6 (PMC7118094; doi:10.1038/s41598-020-62847-6)
Supplement: Supplementary file 1 — Supplementary Information. [file 41598_2020_62847_MOESM1_ESM.pdf]

Supporting information

Crystallographic characterization of a tri-Asp metal-binding site at the three-fold symmetry axis of LarE

Authors

Matthias Fellner[ab], Kamren G. Huizenga[b], Robert P. Hausinger[bc]* and Jian Hu[bd]*

[a]Biochemistry, University of Otago, PO Box 56, Dunedin, Otago, 9054, New Zealand

[b]Biochemistry and Molecular Biology, Michigan State University, 603 Wilson Road, East Lansing, Michigan, 48824, USA

[c]Microbiology and Molecular Genetics, Michigan State University, 567 Wilson Rd, East Lansing, Michigan, 48824, USA

[d]Chemistry, Michigan State University, 578 S Shaw Ln, East Lansing, Michigan, 48824, USA

*Correspondence email: hausinge@msu.edu; hujian1@msu.edu

Table S1

Macromolecule production information

Source organism	*Lactobacillus plantarum* (strain ATCC BAA-793 / NCIMB 8826 / WCFS1) (strain: ATCC BAA-793 / NCIMB 8826 / WCFS1)
Expression vector	pNZ8048 *Lactococcus lactis* plasmid, pBADHisA *Escherichia coli* plasmid
Expression host	*L. lactis* NZ3900 or *E. coli* Arctic-Express cells
Complete amino acid sequence of the construct produced	MATLATKKATLVAALKDLQRVTVAFSGGID STLVLKMALDVLGRDNVTAVVANSELFTDEE FDKAMSLAEELGANVQGTT LDYLSDDHIKNNTPDSWYYAKKMFYSRLND IAANNGSAAVLDGMIKNDENDYRPGLKARSE AGARSLLQEADFFKTDVRA LAQELGLTNWNKVASCSVSSRFPYGTTLTH DNIAQVMAAEKYLRSLGFPTVRVRFHNDIAR IELPEARIGDFLVFNDRVN RQLQSLGFRYVTLDLGGFRSGRMNDTLTKA QLATFAASWSHPQFEK

Table S2

LarE crystallization conditions. Hanging (H)- or sitting (S)-drop vapor diffusion method. Drop size is shown as µL protein + µL reservoir. Protein concentrations are in mg/mL before mixing. Highest resolution for each condition is indicated (Å). The space group (SG) for each condition, as well as the number (Nr) of subunits in the asymmetric unit are indicated. Conditions marked with * were used for the Hampton Additive ScreenTM co-crystallization screen.

H	µl+µl	mg/mL	Condition; for deposited structures PDB IDs listed	Å	SG	Nr
Optimal condition for reproducibility						
H	5+5	20-30	0.05 M Bis-Tris, pH 6.5, 0.1 M $(NH_4)_2SO_4$, 30% v/v PE (15/4 EO/OH)*	2.1-2.8	P4122	6
Variations of the optimal conditions						
H	4+4/5+5	23-39	0.05 M Bis-Tris, pH 6.5, 0.05-0.15 M $(NH_4)_2SO_4$, 27.5-37.5% v/v PE (15/4 EO/OH); 5UDQ, 5UDR, 5UDS, 5UDV, 5UDW, 6B2M, 6B2O, 6UTQ, 6UTR and 6UTT	2.09	P4122	6
H	5+5	30	0.05 M Bis-Tris, pH 6.5, 0.1 M $(NH_4)_3PO_4$, 35% v/v PE (15/4 EO/OH), 5UDX	2.24	P4122	6

H	5+5	30	0.05 M HEPES, pH 7.0, 0.1 M $(NH_4)_2SO_4$, 25-35% v/v PE (15/4 EO/OH); 5UDU	2.60	P4122	6
H	0.6+1.2	25	0.05 M MOPS, pH 7.0, 0.1 M (NH4)2SO4, 25% v/v PE (15/4 EO/OH); 6UTP	3.55	P4122	6
H	5+5	30	0.05 M Bis-Tris, pH 6.5, 0.1 M Li_2SO_4, 35% v/v PE (15/4 EO/OH)	2.60	P4122	6
H	5+5	30	0.05 M Bis-Tris, pH 6.5, 0.1 M NH_4Cl, 35% v/v PE (15/4 EO/OH)	3.10	P4122	6
H	5+5	30	0.11 M Bis-Tris, pH 6.5, 2 m*M* sulfosuccinic acid, 35% v/v PE (15/4 EO/OH)	3.20	P4122	6
H	5+5	30	0.05 M Bis-Tris, pH 6.5, 0.02 M sodium thiosulfate, 35% v/v PE (15/4 EO/OH)	2.36	C2221	12
Other conditions resulting in determined datasets						
H	5+5	25	21% v/v Tacsimate, pH 6.1*; 5UNM	2.58	P4122	6
S	0.5+0.5	20	0.2 M sodium malonate, 20% w/v PEG 3,350	2.61	P121	6
S	0.5+0.5	13	0.1 M imidazole, pH 7.0, 0.15 M Malic acid, pH 7.0, 22% PEG monomethyl ether	2.66<br>3.10	C2221<br>R3	12<br>8
S	0.5+0.5	20	0.1 M HEPES, pH 7.5, 0.2 M ammonium acetate, 25% w/v PEG 3,350	2.79	P121	6
H	4+2	21	28% v/v Tacsimate, pH 6.5, 4 m*M* ATP, 10 m*M* $MgCl_2$, 5UDT	3.19	P3122	6
S	0.5+0.5	20	0.1 M citric acid, pH 6.5, 20% w/v PEG 6,000	3.73	P121	6
Additional initial screening hits						
S	0.5+0.5	20	0.1 M HEPES, pH 7.0, 5% v/v Tacsimate, pH 7.0, 10% w/v PEG monomethyl ether 5,000	~3.5		
S	1+1	9	0.1 M Imidazole, pH 8.0, 1 M ammonium phosphate	~4		
S	1+1	23	0.1 M Tris, pH 7.0, 0.2 M $MgCl_2$, 10% w/v PEG 8,000	~5		
S	2+1	23	0.1 M Bis-Tris, pH 5.5, 1.17 M $(NH_4)_2SO_4$, 1.0% w/v PEG 3,350	~7		
S	2+1	25	0.2 M potassium sodium tartrate, 18.5% w/v PEG 3,350	~7		

S	0.5+0.5	20	0.1 M HEPES, pH 7.5, 10% w/v PEG 8,000, 8% v/v ethylene glycol	~7		
S	2+1	23	0.1 M MES, pH 6.0, 3.8% w/v PEG 3,350	~8		
S	2+1	24	0.1 M Tris, pH 8.5, 0.2 M Li_2SO_4, 28.0% w/v PEG 3,350	~8		
S	0.5+0.5	18	0.1 M potassium/sodium phosphate, pH 6.2, 10% w/v PEG 3,000	~8		
S	2+1	16	0.1M Tris, pH 8.5, 1.5 M ammonium sulfate, 12% v/v glycerol	~8		

Table S3

LarE structures

LarE structure	PDB ID	Resolution (Å)	Reference
Apo form	5UDQ	2.09	[1]
Nicotinamide mononucleotide bound	5UDR	2.62	[1]
MgATP bound	5UDS	2.37	[1]
AMP bound	5UDT	3.19	[1]
Mn bound	5UDU	2.79	[1]
Fe bound	5UDV	2.62	[1]
Ni bound	5UDW	2.70	[1]
Zn bound	5UDX	2.78	[1]
Apo form with flexible loop	5UNM	2.58	[1]
Coenzyme A bound	6B2M	2.09	[2]
C176A variant apo form	6B2O	2.35	[2]
Ca bound	6UTT	2.49	This publication
Co bound	6UTP	3.55	This publication
Cu bound	6UTR	2.44	This publication
Cd bound	6UTQ	2.39	This publication

Table S4

Effective ionic radii in pm of selected elements investigated in this study

Name	Ionic radii	Tri-Asp binding
Cr^{3+}	61.5	No
Fe^{3+}	64.5	Yes
Ni^{2+}	69	Yes
Mg^{2+}	72	No
Cu^{2+}	73	Yes
Zn^{2+}	74	Yes
Co^{2+}	74.5	Yes
Fe^{2+}	78	Yes
Mn^{2+}	83	Yes
Y^{3+}	90	No
Cd^{2+}	95	Yes
Ca^{2+}	100	Yes
Sr^{2+}	118	No
Ba^{2+}	135	No

Table S5

Investigated element coordination number # in MetalPDB. The most frequent coordination is shaded grey.

	#1	#2	#3	#4	#5	#6	#7	#8	#9	#10	Tri-Asp binding
Ca	440	487	572	662	688	926	967	230	12	1	Yes
Mn	140	134	144	160	199	423	54	8	0	0	Yes
Fe	45	66	78	411	185	531	33	15	9	6	Yes
Co	58	48	58	71	103	189	14	2	0	2	Yes
Ni	113	171	115	161	130	174	13	6	1	0	Yes
Cu	59	97	123	133	76	33	8	0	0	0	Yes
Zn	534	910	1006	2056	793	477	78	11	0	0	Yes
Cd	260	384	389	412	271	220	72	11	1	1	Yes
Cr	1	1	0	1	1	6	0	0	0	0	No
Mg	1499	1315	1183	1210	1102	2255	164	24	5	2	No
Y	13	22	9	11	12	10	6	3	4	0	No
Sr	21	24	27	25	16	25	17	12	0	0	No
Ba	30	34	19	16	7	8	0	2	1	0	No

References

1 Fellner, M., Desguin, B., Hausinger, R. P. & Hu, J. Structural insights into the catalytic mechanism of a sacrificial sulfur insertase of the N-type ATP pyrophosphatase family, LarE. *Proc Natl Acad Sci U S A* **114**, 9074-9079, doi:10.1073/pnas.1704967114 (2017).

2 Fellner, M., Rankin, J. A., Desguin, B., Hu, J. & Hausinger, R. P. Analysis of the Active Site Cysteine Residue of the Sacrificial Sulfur Insertase LarE from *Lactobacillus plantarum*. *Biochemistry* **57**, 5513-5523, doi:10.1021/acs.biochem.8b00601 (2018).
